# The relationship between perceived teacher support and student engagement in Chinese senior high school English classrooms: the mediating role of learning motivation

**DOI:** 10.3389/fpsyg.2025.1563682

**Published:** 2025-03-05

**Authors:** Wenxing Zhang, Jiayue Hu

**Affiliations:** School of Foreign Languages, Jiangxi Science and Technology Normal University, Jiangxi, China

**Keywords:** perceived teacher support, student engagement, learning motivation, mediation, Chinese senior high school

## Abstract

**Introduction:**

In the current educational context, student engagement serves as a key indicator of both teaching quality and overall educational effectiveness in senior high school education.

**Methods:**

This study employed structural equation modeling to analyze the self-report questionnaire data from 314 Chinese senior high school students, aiming to examine the relationship between perceived teacher support and student engagement in English classrooms as well as the mediating role of learning motivation (intrinsic motivation and extrinsic motivation).

**Results:**

The results indicate that perceived teacher support, learning motivation, and student engagement in English learning are generally moderate to high. There is a positive correlation between teacher support, learning motivation, and student engagement. Teacher support significantly predicts student engagement yet learning motivation plays a mediating role in this relationship, with intrinsic motivation having a greater mediating effect than extrinsic motivation.

**Discussion:**

These findings provide valuable insights into how teacher support influences student motivation and engagement, offering practical strategies for improving instructional approaches in senior high school English education.

## 1 Introduction

With the development of globalization, English, as an important tool for international communication, has become increasingly important. The openness of English learning is not only reflected in the universality and diversity of language knowledge, but also reflected in the cultivation of cross-cultural understanding, critical thinking, and self-regulated learning. The openness of English learning provides students with a broad learning space and rich learning resources, which helps to stimulate students' interest and motivation, to promote student engagement in English learning activities. Student engagement in learning is recognized as a key factor in English learning, where increased engagement in learning is critical to improving the quality of teaching and learning achievements. During the senior high school stage, teachers significantly influence student learning as classroom instructors and extracurricular designers. Positive teacher-student interactions have the potential to impact students' academic emotions, learning behavior patterns, and academic achievements (Engels et al., 2021; Longobardi et al., 2021). Therefore, the more teacher support students perceive, the higher the level of student engagement (Strati et al., 2017). However, previous studies have highlighted the positive impact of perceived teacher support on student engagement, but most have focused on higher education (Sadoughi and Hejazi, 2021; Xu et al., 2023b), leaving a gap in understanding the situation for Chinese senior high school students. Chinese senior high school students face unique educational environments and pressures brought by the exam-driven learning culture, especially the heavy burden of the national college entrance exam. In this environment, the role of teacher support may be more prominent, because it can not only relieve students' academic pressure but also stimulate their learning motivation and promote student engagement.

Given this, the study aims to explore the interaction between perceived teacher support and student engagement in Chinese senior high schools. It seeks to develop a mediation model to reveal the path of learning motivation in this relationship, as well as to propose corresponding strategies to improve student engagement and the quality of senior high school English teaching.

### 1.1 Student engagement

Student engagement refers to a positive state of students' integration into learning tasks and is essentially an important reflection of students' enjoyment of the learning process (Fredricks et al., 2019), which is pivotal in the current educational landscape, especially in English learning, where increased engagement in learning is critical to improving the quality of teaching and achievements. Regarding the construction of concept, the three-dimensional model (Fredricks et al., 2004) has been recognized by many scholars and widely applied in empirical studies, encompassing behavioral, cognitive, and emotional engagement. Behavioral engagement relates to the time and effort students put into learning tasks and extracurricular activities, such as individual efforts, teacher-student interaction, and peer interaction. Emotional engagement focuses on positive relationships between students and their peers and teachers (Xu et al., 2023a). Cognitive engagement highlights the mental processing of knowledge and the cognitive and metacognitive strategies learners employ (Xu et al., 2024).

Recently, the notion of student engagement has gained prominence in second language education (Hiver et al., 2024). As a dynamic and variable state, student engagement is influenced by both personal traits and external environments (Fredricks et al., 2004). Scholars have extensively studied external environments influencing student engagement, such as social support, teacher support, and peer support (Luan et al., 2023; Sadoughi and Hejazi, 2021; Xerri et al., 2018). During the senior high school stage, teachers significantly influence student learning the more teacher support students perceive, the higher the level of student engagement (Strati et al., 2017). Sulis and Philp (2021) explored the relationship between the learning environment and student engagement in foreign language learning. They discovered that positive peer-to-peer and teacher-student relationships can influence one's decisions to participate in learning activities. De Barba et al. (2016) explored how individual characteristics predict student engagement. They found that students' motivation positively affects their learning interests, engagement, and performance.

In conclusion, student engagement interacts with various personal and environmental factors in the context of Chinese senior high schools. However, in order to improve students' psychological wellbeing in English learning, boost their engagement, and ultimately improve their English performance, a deeper understanding of these correlations is imperative.

### 1.2 Perceived teacher support and student engagement

Based on the ecological systems theory, school is a micro-system that significantly influences students' development. Teachers play a key role in developing students' motivation and academic performance (Skinner et al., 2008; Xu et al., 2023b). Teacher support is the main social support that learners receive in the school. Social support in the school environment is one of the most important classroom environment factors, which is to help and care for learners' academic study and spirit, and will affect learners' academic performance, physical and wellbeing, and how to conduct constructive management of stress (Ghaith, 2002). Based on the self-determination theory, effective teacher support is viewed as being cognitive, emotional (Skinner and Belmont, 1993), or autonomy-focused (Lei et al., 2018). Teacher support includes emotional, autonomy, and cognitive support (Chai and Gong, 2013). Autonomy support refers to teachers granting students freedom during learning activities (Reeve, 2009), nurturing their needs, interests, and offering classroom opportunities for students to direct their learning using their motivations (Reeve, 2024). Cognitive support fosters cognitive development through intellectual challenges, learning strategies, and resources. Emotional support provides students with positive attention, emotional connection, and stress management support. Students who perceive autonomy show greater curiosity and desire to challenge and engage more seriously in learning activities (Deci and Ryan, 2000).

Existing studies have investigated how teacher support affects student engagement, and most of them have focused on the science and math domains (Liu et al., 2018; Tas et al., 2019). Research on the relationship in English environments has only emerged in the past few years. According to Sadoughi and Hejazi (2021), teacher support exerts a notable impact on Iranian university students' engagement in English learning. Wentzel et al. (2016) pointed out that students' perceived teacher support plays a more critical role in their engagement and effort than parental support and peer support. Teacher support has a positive impact on student engagement in online English learning (Luan et al., 2023). In addition, existing studies have found that teacher support may be associated with student engagement through the internal factors of learners, and then have an impact on student engagement (Helgeson and Lopez, 2010). Therefore, it is important to delve into the potential mechanism by which teacher support influences student engagement.

### 1.3 Learning motivation and student engagement

Research has shown that both learning motivation and academic emotions are important variables running through an individual's learning process, and most of the existing studies have centered on the effect of academic emotions on student engagement (Dewaele and Li, 2021; Gunness et al., 2023), yet the issue of the relationship between motivation and learning engagement is less addressed, and it is found that motivation is more predictive of student engagement than emotions (Fried and Chapman, 2012). As one of the key elements influencing the success of learning a second language, motivation is considered to be a complex component of an individual's psychological and behavioral processes, influencing their level of engagement, emotional experiences, and persistence in specific tasks (De Barba et al., 2016; Dörnyei, 1998). Self-determination theory views motivation as a continuum that progresses from amotivation to extrinsic motivation and then intrinsic motivation, with a gradual increase in the degree of self-determination (Deci and Ryan, 2000). Extrinsic motivation refers to engaging in behaviors for instrumental reasons to attain other benefits (Deci and Ryan, 2000). Based on varying degrees of autonomy in the decision-making and regulation of behavioral activities, self-determination theory further categorizes extrinsic motivation into four types: external, introjected, identified, and integrated regulation. Intrinsic motivation, stemming from intrinsic interest and enjoyment, has the highest autonomy (Deci and Ryan, 2000). Integrated regulation is often considered together with intrinsic motivation in research due to their close relationship (Joe et al., 2017).

Focusing on the interrelationship between learning motivation and student engagement, researchers have explored the impact of intrinsic and extrinsic motivation on student engagement from the perspective of the dualistic model of motivation. Most scholars tend to believe that learning activities are predominantly individual and self-directed, and strong intrinsic motivation helps learners better engage in learning activities. In the context of English learning, intrinsic motivation is generally considered to be more conducive to engagement as it prompts learners to pursue knowledge out of personal interest and enjoyment rather than being motivated by external rewards or stress. They have found that successful students exhibit strong intrinsic motivation (Huett et al., 2008). Studies by Reeve and Tseng (2011) and Reeve and Lee (2014) have demonstrated that intrinsically motivated students are more likely to be actively engaged in their learning and exhibit higher levels of curiosity. However, the literature presents mixed findings regarding how extrinsic motivation affects student engagement. Some studies suggest that strong extrinsic motivation tends to evoke a psychological tendency among learners to avoid online learning, largely inhibiting their engagement in online learning (Cho and Heron, 2015). Walker et al. (2006) found that students mainly motivated by external rewards demonstrated less intrinsic interest in the task and were less likely to have deep engagement with the material. Similarly, Saeed and Zyngier (2012) revealed that students focusing on grades and external validation tended to exhibit a superficial learning strategy and lacked a genuine connection to the content. Some scholars argue that while intrinsic motivation is more stable and enduring, extrinsic motivation can also effectively promote student engagement (Renninger and Hidi, 2015). Uyen (2016) found that teachers can design classroom teaching activities to adjust students' identification with the importance and relevance of learning tasks driven by extrinsic motivation, which can also contribute to increasing students' engagement in classroom learning. When extrinsic rewards were aligned with students' personal goals and interests, they could enhance motivation and engagement. Similarly, Laili and Nashir (2021) suggested that extrinsic motivation could improve online learning engagement, yet there is still no consensus between the researchers on the relationship between external motivation and student engagement. Regarding different views on external motivation, cultural and educational contexts play an important role. In some cultural and educational systems, extrinsic rewards are seen as recognition and encouragement, which can enhance motivation and participation. However, in other cases, they may be seen as stress or a means of control. In addition, the classroom environment and teaching practice also play a crucial role. Teachers designing classroom activities that adjust the importance and relevance of students' recognition and learning tasks can effectively promote learning engagement.

Therefore, further exploration is required to comprehend the link between various types of learning motivation and student engagement within Chinese senior high school English classrooms. In addition, the results of existing empirical studies have emphasized the influence of learning motivation on student engagement, the role of learning motivation in mediating the relationship between external factors and student engagement requires further investigation.

### 1.4 Mediating role of learning motivation

According to self-determination theory and ecological systems theory, student engagement is dynamic (Hiver, 2022; Sulis, 2024), and is influenced by various factors, such as the learning environment, teachers, peers, and learners themselves. Among these factors, teachers emerge as a pivotal influence on student engagement with learning motivation consistently being identified as a key influence on student engagement (An et al., 2022). Teacher support positively predicts learning motivation among senior high school students (Affuso et al., 2023; Cooper, 2014). Teachers also play an indispensable role in students' learning processes, and by providing appropriate support, teachers can establish positive teacher-student relationships and foster the internalization of motivational behaviors, consequently enhancing student engagement in learning (Niemiec and Ryan, 2009). Furthermore, Chen and Kraklow (2015) further demonstrated that motivation is a significant predictor of English learning engagement among Chinese college students.

In summary, existing studies show a correlation between perceived teacher support, student engagement, and learning motivation (An et al., 2022; Chiu et al., 2024; Strati et al., 2017). However, the mechanisms of learning motivation between these factors are not fully understood. Specifically, more studies are required to determine how intrinsic and extrinsic motivation mediate the relationship between perceived teacher support and student engagement. Therefore, this study aimed to explore the potential relationship between these three variables, with a particular emphasis on the mediating role of learning motivation. The following questions will be addressed:

1. What are Chinese senior high school students' levels of perceived teacher support, learning motivation, and student engagement in English learning?
2. What are the direct relationships between Chinese senior high school students' perceived teacher support, learning motivation, and student engagement in English learning?
3. Does the learning motivation of Chinese high school students mediate the relationship between their perceived teacher support and student engagement in English learning? if yes, to what extent?

## 2 Methods

### 2.1 Participants

This study employed a quantitative research design, which is a survey study, where quantitative data of research subjects are collected through a questionnaire survey and then quantitative analysis of the research questions. This study employed various sampling methods. It initiated with stratified sampling, based on the educational resources or quality of schools. Schools were divided into two strata: those located in urban areas with more developed educational systems, and those in counties with relatively less developed educational resources. From each stratum, two schools were randomly selected from a province in southeastern China. Subsequently, students were selected randomly within these four schools.

Reasonable sample size is essential during structural equation model analysis. According to the sample size estimation principle of the structural equation model, the ideal sample size should be 5–10 times the number of model variables. In this study, the questionnaire designed 26 model variables, therefore, to ensure the robustness of the model analysis, Barrett (2007) stated that the sample size needs to reach at least 200 participants. In addition, Barrett argued that the structural equation model generally uses the built-in maximum likelihood method, which severely inflates chi-square values at sample sizes >500. This can lead to a poor fit of the model (Barrett, 2007). Therefore, when planning the sample size, we need to find a balance point to ensure sufficient sample size to improve the analysis's robustness and avoid the model fitting problem caused by excessive sample size. Considering both the sample loss and the invalid questionnaires, we have reserved an additional sample size of about 20% in the planning phase, yet the reality was that there are always some respondents who do not answer or the answer is invalid. To compensate for this loss part, we need to increase the proportion to the calculated sample size. Considering the above factors, the final estimated sample size is 349.

The exclusion criteria for invalid questionnaires were selecting different options for repeated questions and providing the same answer for all questions. Eleven questionnaires were excluded because all responses were selected using the same option. Twenty-four questionnaires were excluded because different answers were given for repeated questions. A total of 314 questionnaires remained, which led to an approximate response rate of 90%. The sample included 152 male students (48.3%) and 162 female ones (51.7%). Promoting study participation is vital, which we achieved by clearly communicating its purpose, guaranteeing anonymity and confidentiality, and providing incentives such as gift cards. This created a secure, motivating atmosphere for potential participants. All participants were clearly informed about the purpose of the study. All of them voluntarily participated and actively cooperated with this study. Meanwhile, to avoid comprehension bias, all statements in the questionnaire were provided in Chinese.

### 2.2 Measures

This study used a questionnaire composed of four parts: the informed consent, the perceived English teacher support questionnaire, the student engagement questionnaire, and the English learning motivation questionnaire. All of the above questionnaires were measured using a five-point Likert scale, with responses ranging from 1 (“completely disagree”) to 5 (“completely agree”).

#### 2.2.1 Perceived English teacher support questionnaire

This questionnaire was adapted and revised from the teacher support scale (Chai and Gong, 2013), specifically designed with Chinese senior high school students as the research participants, aligning perfectly with the age group of our study participants and research objectives. It incorporates the unique characteristics of English language learning among Chinese senior high school students and has undergone extensive validation within the Chinese educational context. The scale divides teacher support into three dimensions: autonomy support (three items, e.g., “My English teacher gives us enough time to study on our own or think independently.”), cognitive support (three items, e.g., “My English teachers will provide us with a wealth of information and learning methods.”), and emotional support (four items, e.g., “My English teacher understands and cares about me.”), with a total of 10 items.

#### 2.2.2 Student engagement questionnaire

This questionnaire was developed by combining the student engagement scale (Ren, 2021) and the student engagement instrument scale (Fredricks and McColskey, 2012) while taking into account the educational background and current situation in China. Given that the time gap between the compilation of this questionnaire and our current usage is within 3 years, it introduces minimal bias and is highly suitable for our research. Moreover, both original scales specifically focus on English learners as their research subjects, making this questionnaire particularly relevant to our research participants. To avoid duplication, redundant items measuring the same structure in a similar or the same way will be removed. Finally, it contains 10 items, which are divided into three dimensions: behavioral engagement (three items, e.g., “I always answer the teacher's questions in English class.”), emotional engagement (three items, e.g., “It is enjoyable to learn new things in the English classroom.”), and cognitive engagement (three items, e.g., “I can focus on a deeper understanding of what I am learning in the English learning process.”).

#### 2.2.3 English learning motivation questionnaire

The questionnaire was adapted from the Language Learning Orientation Scale (Noels et al., 2000). The revised scale was adapted for Chinese students and was reported to have good reliability and validity. The revised questionnaire was adapted for Chinese students and was reported to have good reliability and validity (Lou and Noels, 2021), with prior empirical success. The scale consists of two dimensions: extrinsic motivation (external regulation, introjected regulation, identified regulation) and intrinsic motivation, which consists of three items for intrinsic motivation (e.g., “I study hard in English because I like the language.”) and three items for extrinsic motivation (e.g., “I think learning English well can make me more competitive in job hunting.”).

The study included two implementation phases: pilot study and formal study. Eighty-one Chinese senior high school students participated in the pilot study, which showed that the standardized Cronbach's alpha for the three scales was 0.78, 0.83, and 0.76, respectively; and the validity test KMO measures were 0.80, 0.84, and 0.69, with Barlett's spherical test significant (*p* < 0.001), indicating that the sample data were suitable for exploratory and confirmatory factor analyses. An exploratory factor analysis was conducted on the learning motivation scale. Four items were removed because of the low factor loading (0.29, 0.42, 0.39, and 0.54), which affected the factors' explanatory power. After deletion, the cumulative explained variance increased significantly. The principal component analysis method was used to extract factors with characteristics >1. In total two factors were extracted, and the two factors explained 69.512% of the original variance, resulting in the formal scale of this study, which consisted of three items for intrinsic motivation and three items for extrinsic motivation. The pilot study showed that these scales had good reliability and validity.

In the formal study, the standardized Cronbach's alpha for the three questionnaires were 0.89, 0.86, and 0.76, respectively; and the validity test KMO measures were 0.90, 0.89, and 0.74, with Barlett's spherical test significant (*p* < 0.001). The validation factors analyses showed that the values of the indicators of the three questionnaires were within the acceptable range[χ^2^/*df* = 2.71 (< 3), CFI = 0.96 (>0.9), TLI = 0.94 (>0.9), GFI = 0.95 (>0.9), RMSEA = 0.07 (< 0.08), SRMR = 0.04 (< 0.08); χ^2^/*df* = 2.16, CFI = 0.96, TLI = 0.95, GFI = 0.96, RMSEA = 0.06, and SRMR = 0.05; χ^2^/*df* = 2.59, CFI = 0.98, TLI = 0.96, GFI = 0.98, RMSEA = 0.07, SRMR = 0.07], the structural model fit was good and had good structural validity (Hu and Bentler, 1999). All questionnaires have good internal consistency with good reliability and validity.

### 2.3 Data collection and analysis

The survey was administered with the authorization of head teachers, English teachers, and the students. Paper-based questionnaires were distributed to students by class. Before issuing the questionnaire, the teachers explained the purpose and requirements of the questionnaire to the students. All the questionnaires were administered uniformly in the classroom or during the evening study sessions. Participants were asked to complete the questionnaire independently within 10 min. Teachers supervised the whole process to ensure that students would fill in truthfully, and prevent discussions between the students, then the completed questionnaires were collected immediately. After the questionnaire was manually entered, the SPSS 26.0 and Amos 26.0 were utilized for data analysis.

Firstly, the Harman single-factor test was conducted to assess common method bias. Secondly, normal distribution tests were performed using SPSS 26.0. Thirdly, descriptive statistics were calculated using SPSS 26.0 to explore the characteristics of teacher support, learning motivation, and student engagement. The association between these variables was investigated using Pearson correlation analysis. Fourth, Amos 26.0 was employed to construct a structural equation model to examine the mediating effects of two forms of learning motivation (intrinsic motivation and extrinsic motivation) on the relationship between teacher support and student engagement. Lastly, the significance of mediating effects was tested using bootstrap methods with strong standard errors (Field, 2017). Using 5,000 resampled data points, the bootstrap method produced 95% deviation-corrected confidence intervals. If there are no zeros in the confidence interval, the significance of the indirect effects is indicated. All statistical tests were two-tailed.

## 3 Results

### 3.1 Common method bias test

As the data collected in this study was predominantly self-reports by the participants, there is a possibility that common method bias could affect the results. To evaluate and test for common method bias, the researcher employed Harman's single-factor test (Podsakoff et al., 2003). A total of 26 items representing the three research variables were included in the exploratory factor analysis of SPSS 26.0, and all items were analyzed by extracting one common factor using principal component analysis. The results show that there are five factors >1. The first factor explains 32.96% of the total variances, which is below the critical threshold of 40%. These findings indicate that there is no significant common method bias in the sample data used in this study. However, it is imperative to acknowledge that Harman's single-factor test may not adequately eliminate bias. Therefore, the interpretation of these results must be approached with caution.

### 3.2 Descriptive statistics and correlation analyses

This study was analyzed using SPSS 26.0, Table 1 displays the results of the descriptive analyses of the quantitative data along with the normal distribution test. The findings indicated that the absolute values of skewness and kurtosis for each variable are below 1, confirming that data follows a normal distribution overall.

**Table 1:** Descriptive statistics and correlation analysis (*N* = 314).

**Variables**	**(1)**	**(2)**	**(3)**	**(4)**	**(5)**	**(6)**	**(7)**	**(8)**	**(9)**	**(10)**	**(11)**
(1) ES	1										
(2) AS	0.638^**^	1									
(3) CS	0.579^**^	0.674^**^	1								
(4) PTS	0.936^**^	0.868^**^	0.681^**^	1							
(5) IM	0.409^**^	0.281^**^	0.254^**^	0.392^**^	1						
(6) EM	0.306^**^	0.341^**^	0.361^**^	0.353^**^	0.332^**^	1					
(7) LM	0.421^**^	0.366^**^	0.357^**^	0.439^**^	0.849^**^	0.741^**^	1				
(8) BE	0.408^**^	0.269^**^	0.296^**^	0.385^**^	0.518^**^	0.409^**^	0.559^**^	1			
(9) EE	0.450^**^	0.408^**^	0.435^**^	0.477^**^	0.607^**^	0.442^**^	0.651^**^	0.589^**^	1		
(10) CE	0.378^**^	0.288^**^	0.267^**^	0.375^**^	0.658^**^	0.423^**^	0.659^**^	0.584^**^	0.624^**^	1	
(11) SE	0.481^**^	0.372^**^	0.387^**^	0.480^**^	0.688^**^	0.495^**^	0.723^**^	0.868^**^	0.852^**^	0.846^**^	1
Mean	3.694	3.911	3.881	3.787	3.361	3.762	3.633	3.576	3.640	3.414	3.546
SD	0.906	0.854	0.893	0.802	1.056	0.817	0.783	0.836	0.926	0.904	0.756
Skewness	−0.402	−0.739	−0.696	−0.498	−0.499	−0.901	−0.615	−0.395	−0.592	−0.245	−0.327
Kurtosis	−0.322	0.207	0.920	−0.055	−0.392	0.793	−0.296	−0.224	−0.026	−0.343	−0.392

ES, Emotional support; AS, Autonomy support; CS, Cognitive support; PTS, perceived teacher support; IM, Intrinsic motivation; EM, Extrinsic motivation; LM, learning motivation; BE, Behavioral engagement; EE, Emotional engagement; CE, Cognitive engagement; SE, student engagement.

^**^p < 0.01.

Senior high school students reported a high level of overall teacher support (*M* = 3.787, *SD* = 0.802). While cognitive (*M* = 3.881) and autonomy support (*M* = 3.911) were high, emotional support was comparatively lower (*M* = 3.694). The mean student engagement was 3.546 (*SD* = 0.756), behavioral engagement (*M* = 3.576) and cognitive engagement (*M* = 3.414) were moderate, while emotional engagement (*M* = 3.640) was higher. Moreover, students' English learning motivation (*M* = 3.632, *SD* = 0.783) was generally higher than average, while intrinsic motivation (*M* = 3.361) was medium, and extrinsic motivation (*M* = 3.762) was relatively high.

Based on the normality test result, a correlation analysis was conducted. As shown in Table 1, we found that perceived teacher support, learning motivation, and student engagement were positively and significantly correlated (0.439 < *r* < 0.723, *p* < 0.01). This finding suggests that as Chinese senior high school students perceive greater teacher support in English learning, their learning motivation increases, subsequently enhancing their engagement in English learning.

### 3.3 Analysis of the mediating effect of learning motivation

**Figure 1:**
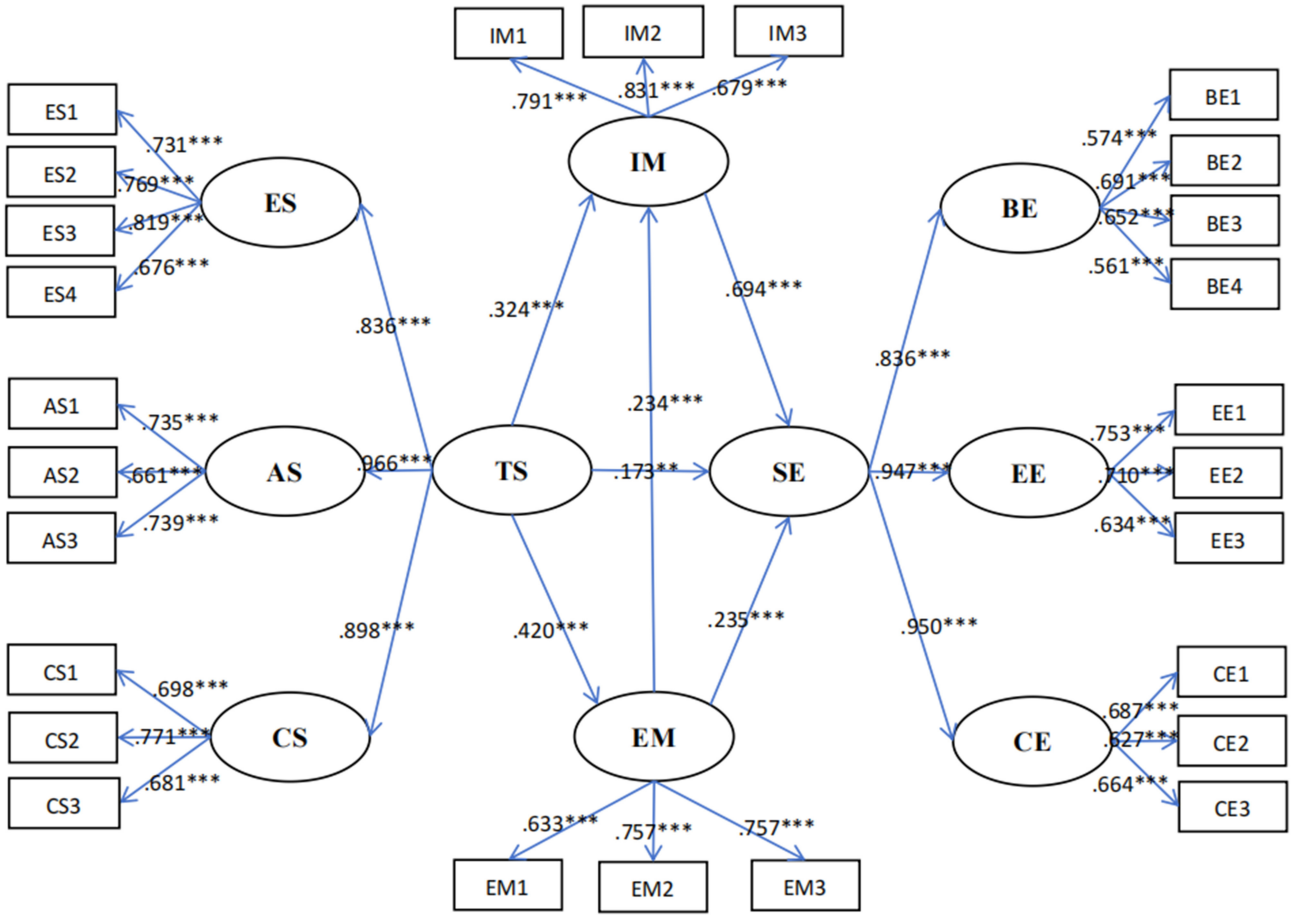
Path diagram of the mediation mode. ES, Emotional support; AS, Autonomy support; CS, Cognitive support; PTS, perceived teacher support; IM, Intrinsic motivation; EM, Extrinsic motivation; LM, learning motivation; BE, Behavioral engagement; EE, Emotional engagement; CE, Cognitive engagement; SE, student engagement. ***p < 0.001, **p < 0.01.

In this study, a structural equation model for parameter estimation using Amos 26.0 was run to examine the mediating effect of learning motivation (intrinsic motivation and extrinsic motivation). Perceived teacher support was set as the predictor variable, English learning motivation (intrinsic motivation and extrinsic motivation) as the mediator, and English student engagement as the outcome variable. The result indicated that the mediating effect model fit the data well (χ^2^/*df* = 1.83, GFI = 0.89, CFI = 0.93, TLI = 0.92, IFI = 0.93, RMSEA = 0.05, SRMR = 0.07), align with recommended thresholds (Hu and Bentler, 1999). In addition, the bootstrap methods with robust standard errors were applied to test the significance of mediating effects (Field, 2017). A bias-corrected bootstrap test (5,000 iterations) was performed to check whether the indirect paths shown in Figure 1 were significant. In the test, the 95% confidence interval of the indirect path coefficient did not include 0, indicating statistical significance.

Table 2 revealed that in the context of Chinese senior high school English classrooms, perceived teacher support had a positive effect on student engagement (β = 0.173, *p* < 0.01). Perceived teacher support significantly predicted learners' intrinsic motivation (β = 0.324, *p* < 0.001), and intrinsic motivation (β = 0.694, *p* < 0.001) positively predicted student engagement. Furthermore, perceived teacher support also significantly predicted learners' extrinsic motivation (β = 0.420, *p* < 0.001), and extrinsic motivation positively predicted student engagement (β = 0.235, *p* < 0.001).

**Table 2:** Results of path relationship test for structural equation modeling.

**Pathway**	**Estimate**	**S.E**.	**Value of *Z***	**C.R**.	***p***
IM ← TS	0.324	0.108	4.167	4.152	^***^
EM ← TS	0.420	0.132	5.417	5.436	^***^
SE ← EM	0.235	0.135	3.750	3.712	^***^
SE ← IM	0.694	0.329	7.477	7.429	^***^
SE ← TS	0.173	0.048	2.917	2.907	0.004^**^

TS, teacher support; IM, intrinsic motivation; EM, extrinsic motivation; SE, student engagement.

^**^p < 0.01, ^***^p < 0.001.

As shown in Table 3, the direct effect size of perceived teacher support on student engagement was 0.173, the 95% confidence interval did not contain 0 (0.040, 0.309), accounting for 30.62% of the total effect. In terms of indirect effects, the indirect effect of teacher support on student engagement through intrinsic motivation was 0.225 and the 95% confidence interval did not contain 0 (0.108, 0.352), 39.82% of the total effect. Similarly, the mediation effect of extrinsic motivation also showed a significant result, with a coefficient of 0.099, and the 95% confidence interval for the mediation coefficient not containing 0 (0.044, 0.172), 17.52% of the total effect. Regarding the indirect effect of teacher support on student engagement through both intrinsic motivation and extrinsic motivation, the 95% confidence interval (from 0.018 to 0.128) indicated that it was also significant. The effect size was 0.068, accounting for 12.04% of the total effect. Therefore, learning motivation plays a partially mediating role in the relationship between perceived teacher support and student engagement among senior high school students.

**Table 3:** Bootstrap mediation affects test results.

**Pathway**	**Estimate**	**S.E**.	**95% CIs**		***p***	**Relative effect (%)**
			**Lower**	**Upper**		
TS → IM → SE	0.225	0.063	0.108	0.352	^***^	39.82
TS → EM → SE	0.099	0.068	0.044	0.172	^***^	17.52
TS → SE	0.173	0.033	0.040	0.309	0.013^*^	30.62
TS → EM → IM → SE	0.068	0.028	0.018	0.128	0.007^**^	12.04

TS, teacher support; IM, intrinsic motivation; EM, extrinsic motivation; SE, student engagement.

^*^p < 0.05, ^**^p < 0.01, ^***^p < 0.001.

## 4 Discussion

This study first measured the levels of perceived teacher support, learning motivation, and student engagement among Chinese senior high school students. Subsequently, it investigated the relationships between these three factors and further validated the mediating function of learning motivation.

### 4.1 Levels of perceived teacher support, learning motivation, and student engagement

Firstly, the overall level of teacher support was relatively high in Chinese senior high schools. Among the three dimensions, cognitive and autonomy support was highly rated, indicating that senior high school English teachers prioritize enhancing students' English skills, teaching-learning strategies, and fostering academic achievement. However, while cognitive and autonomy support scored impressively, emotional support scored slightly lower, suggesting that there is room for improvement in fostering belonging and happiness in the classroom. Teachers need to strengthen their emotional communication with students by actively listening, acknowledging their emotions, and providing reassurance and encouragement, to narrow perception gaps and ensure that all students feel the care and support of teachers.

The overall level of student engagement was moderate in Chinese senior high schools, aligning with previous research (An et al., 2022). This suggests that, while Chinese senior high school students display a foundational level of engagement in their English learning, there is significant room for further improvement and refinement in their engagement. An in-depth analysis of engagement dimensions revealed that students exhibited relatively strong behavioral and emotional engagement demonstrating great effort and active engagement in classroom activities while maintaining interest in the subject. In contrast, their cognitive engagement was relatively low, with learning strategies in English learning rated as below average. Given these insights, English teachers must focus on teaching and develop effective learning strategies tailored to meet the specific needs of their students.

Regarding learning motivation, students exhibited a moderate-to-high level, demonstrating a certain degree of initiative in English learning. Further examination of intrinsic and extrinsic motivations revealed that extrinsic motivation scored higher than intrinsic motivation, aligning with earlier research (Diseth et al., 2020). The results highlight the motivations of senior high school students during English learning, extrinsic motivation is an important driving force and intrinsic motivation plays a small role and has not yet become the main motivation for learning English. Therefore, English teachers must give priority to motivating and cultivating internal motivation to improve the overall English learning motivation and provide students with stronger learning motivation.

### 4.2 Relationships between perceived teacher support, learning motivation, and student engagement

Secondly, a correlation analysis was conducted between perceived teacher support and student engagement. According to analysis results summarized in Tables 1, 2, it can be seen that perceived English teacher support has a positive correlation with and is positively predictive of student engagement. This result confirms that teacher support is an important external factor in student engagement (Luan et al., 2023; Strati et al., 2017; Sulis and Philp, 2021). In terms of the specific dimensions, all dimensions showed significant positive correlations with learning engagement. The autonomy support (0.966) is the highest, serving as the dominant factor in the construct of teacher support. This indicates that teacher autonomy support has the strongest predictive power on student engagement in English learning (Wang, 2024), validating the effectiveness of self-determination theory within the context of English education (Xu et al., 2024). Compared with primary and middle school students, senior high school students exhibit a stronger need for autonomy, demanding more opportunities to make independent choices and decisions during their learning process. Consequently, teachers respect students' autonomous choices, and independent thinking and provide decision-making opportunities. Students who perceive autonomy exhibit stronger curiosity and a desire for challenge and engage more actively in learning tasks (Deci and Ryan, 2000; Jang et al., 2012). Meanwhile, emotional and cognitive support has predictive power for student engagement, with factor loading values of 0.836 and 0.898, respectively. This suggests that by fostering a positive teacher-student relationship, creating challenging language assignments, offering assistance with learning strategies, and in other ways, English teachers can maximize their students' English learning, process, and experience. A good interactive relationship can enhance students' sense of trust and belonging, promote communication and understanding between teachers and students, and thus improve students' enthusiasm and engagement in learning (Javaid et al., 2024). They can also increase their students' sense of competence and belonging in English language classes and motivate them to actively engage in learning activities.

Regarding the relationship between perceived teacher support and learning motivation, all dimensions of perceived teacher support were substantially correlated with and positively predictive of learning motivation. This finding is consistent with existing studies (Affuso et al., 2023; Chiu et al., 2024; Cooper, 2014). These findings reinforced the concept that teachers play a pivotal role in developing students' academic motivation and engagement, Students with a high degree of teacher support showed higher intrinsic motivation, a more positive learning attitude, and better academic performance (Ahn et al., 2021). Based on self-determination theory, fulfilling the basic psychological needs of individuals for autonomy, competence, and relevance are the key drivers of motivation and behavior, which suggests that teacher support promotes a supportive learning environment that fulfills students' basic psychological needs (Ryan and Deci, 2000). Particularly the need for relatedness, when students perceive their teachers as supportive, they are more likely to feel a sense of belonging and connection in the classroom environments. This sense of relatedness, in turn, serves as a powerful motivator, boosting their motivation to learn and persist in challenging tasks (Wang et al., 2022).

The structural equation model further confirmed that learning motivation, as a comprehensive factor, significantly predicts student engagement, aligning with previous studies (Chen and Kraklow, 2015; Ferrer et al., 2022; Reeve, 2012). Empirical studies have shown that there is a significant positive correlation between learning motivation and learning behaviors, student engagement, and academic achievement (Alamer and Lee, 2019; An et al., 2022; Park and Yun, 2018). When students' learning motivation is stimulated, they are more likely to actively participate in learning activities, engage more time and effort in learning English, and achieve better academic achievements (Wang and Eccles, 2013; Zhang and Zou, 2024). However, the uniqueness of this study lies in its more exhaustive examination of how intrinsic and extrinsic motivations separately influence student engagement. On the one hand, extrinsic motivation can positively predict learning engagement, aligning with the discoveries (Laili and Nashir, 2021; Uyen, 2016), which affirm that extrinsic learning motivation positively impacts students' learning interests, engagement, and academic achievements. Renninger and Hidi (2015) highlighted that extrinsic motivation can be beneficial in structured environments. Furthermore, intrinsic motivation exerts a more potent predictive influence on English learning engagement compared to extrinsic motivation. This finding corroborates the study (Chen and Kraklow, 2015).

### 4.3 Analysis of the mediating effect of learning motivation between perceived teacher support and student engagement

Thirdly, the study explored the mediating function of learning motivation, emphasizing its significant role in motivation in the English learning process. The findings revealed that students perceived teacher support can directly and positively predict student engagement; students' perceived English teacher support affects learning motivation to some extent before it can be transformed into student engagement. Consistent with other studies (Ferrer et al., 2022; Wang and Eccles, 2013), teacher support indirectly influences student engagement through learning motivation. Self-determination theory suggests that motivation and behavior are driven by fulfilling an individual's fundamental psychological needs for autonomy, competence, and relatedness (Ryan and Deci, 2017). When teachers provide support that addresses and fulfills students' basic psychological needs, this support acts as a catalyst in nurturing their learning motivation (Noels et al., 1999), prompting students' learning goals to be directed toward the learning activities themselves. The emergence of learning behavior is to experience pleasure, satisfy curiosity, and seek knowledge from these activities. It serves as the wellspring of students' intrinsic motivation to pursue learning achievement (Ryan and Deci, 2000) and improves students' initiative, engagement, and creativity in learning (Ryan and Deci, 2020; Ruzek et al., 2016). This emphasizes the crucial function of learning motivation in linking external support (e.g., teacher support) with internal behaviors (e.g., engagement). Teacher support fosters learning motivation, which in turn enhances student engagement in English learning. This further confirms that learning motivation acts as a partial mediator between teacher support and student engagement.

In addition, the indirect effect was mainly transmitted through intrinsic motivation (39.82%), and less through extrinsic motivation (17.52%). This indicates the pivotal role of intrinsic motivation in English learning (Chen and Kraklow, 2015; Comanaru and Noels, 2009). Specifically, for Chinese senior high school students, intrinsic motivation is particularly crucial in their English learning, as emphasized by Zhou and Zhou (2018). It primarily stems from students' self-determination and interests, igniting their genuine enthusiasm for English learning, and thereby promoting active engagement. In contrast, extrinsic motivations such as exam pressure, while effective in the short term, are difficult to maintain and may even exert adverse effects on students' mental health and learning interests. Therefore, English teachers must motivate and maintain students' intrinsic motivation by creating a student-centered environment that encourages exploration and discovery. By balancing intrinsic and extrinsic motivations, teachers can more effectively enhance student engagement and help them achieve significant academic achievement. In summary, this study highlights the critical role of learning motivation as a mediator in the relationship. It emphasizes that fostering intrinsic motivation is particularly important for sustaining long-term engagement, while extrinsic motivation, although influential, tends to have a more transient effect. Teacher support, when strategically provided to meet students' psychological needs, can significantly enhance both intrinsic and extrinsic motivation, ultimately leading to higher levels of student engagement and better academic achievements (Wang and Eccles, 2013).

## 5 Conclusion

### 5.1 The findings

The results indicate that the perceived teacher support, learning motivation, and student engagement in English learning are generally at moderate to high levels. There is a positive correlation between teacher support, learning motivation, and student engagement. Teacher support significantly predicts student engagement and learning motivation plays a mediating role in this relationship, with intrinsic motivation having a greater mediating effect than extrinsic motivation.

### 5.2 Theoretical contributions

This study theoretically confirms and further enriches the theories of ecological systems theory and self-determination theory, providing empirical support in the field of second language acquisition for these two theories. This study applies self-determination theory to examine how teacher support influences senior high school students' motivation and engagement, contributing new insights into English language learning. Although existing studies have focused on faculty support, there are relatively few studies on specific subjects (e.g., English). This study will provide new empirical data in this field and deepen the understanding of the working mechanisms of faculty support in different subject contexts.

### 5.3 Practical implications

In addition, the research results reveal the working mechanism of teachers' support and learning motivation in English learning engagement, which has important practical implications for English teaching in Chinese senior high schools. Firstly, teachers should take the initiative to provide sufficient emotional, autonomy, and cognitive support in English teaching. To provide emotional support, teachers can organize various interactive activities such as classroom discussions, group projects, and extracurricular practices to enhance the understanding and trust between teachers and students, fostering a harmonious learning atmosphere. For autonomy support, English teachers should flexibly use teaching methods tailored to course characteristics, students' English proficiency, learning styles, and interests. This includes traditional lecture methods, discussion-based learning, and project-based learning to cultivate students' learning autonomy. Regarding cognitive support, English teachers should prioritize timely interactions initiated by students and provide appropriate responses, encouraging classroom inquiries. Furthermore, teachers should use enlightening language to stimulate students' critical thinking and deep reflections. Secondly, teachers should focus on stimulating students' learning motivation. Teachers can cultivate their intrinsic learning motivation by using diverse teaching methods, creating authentic and engaging learning contexts, and encouraging students to engage in autonomous exploration (Chen et al., 2021). Lastly, teachers should also pay attention to cultivating students' extrinsic motivation by creating a supportive learning environment and giving positive feedback, and encouragement (Bernaus and Gardner, 2008), which can help transform extrinsic motivation into intrinsic motivation, thereby effectively enhancing student engagement in English learning.

### 5.4 Limitations and future directions

However, there are several limitations as well. Firstly, the participants were solely from a province in southeastern China, potentially limiting the broader applicability and generalizability of our findings. To enhance the generalizability of future research, it is crucial to diversify the sample by incorporating students from various geographical regions and educational backgrounds. This will better represent the broader student population and enable the findings to be applied in a wider context. Secondly, the study employed a cross-sectional design, which does not allow for causal interpretations of associations between variables. To overcome this limitation and strengthen causal claims, future research should adopt a longitudinal approach, collecting data from the same participants over an extended period. Additionally, experimental or mixed-method approaches could be used to further investigate the causality between perceived teacher support, student engagement, and learning motivation. Thirdly, relying solely on self-report data from students may introduce social desirability bias and memory recall issues. To validate the relationship between perceived teacher support and student engagement, future research should consider incorporating qualitative methods such as teacher observations and interviews to provide a more comprehensive understanding of the relationships between these variables.

## Data Availability

The original contributions presented in the study are included in the article/Supplementary material, further inquiries can be directed to the corresponding author.
